# Land Use Change Simulation in Rapid Urbanizing Regions: A Case Study of Wuhan Urban Areas

**DOI:** 10.3390/ijerph19148785

**Published:** 2022-07-19

**Authors:** Jinling Zhang, Ying Hou, Yifan Dong, Cun Wang, Weiping Chen

**Affiliations:** 1 Institute of International Rivers and Eco-Security, Yunnan University, Kunming 650091, China; pxyageng@163.com; 2 State Key Laboratory of Urban and Regional Ecology, Research Center for Eco-Environmental Sciences, Chinese Academy of Sciences, Beijing 100085, China; cunwang_st@rcees.ac.cn (C.W.); wpchen@rcees.ac.cn (W.C.); 3 University of Chinese Academy of Sciences, Beijing 101408, China; 4 Yunnan Key Laboratory for International Rivers and Transboundary Eco-Security, Kunming 650091, China

**Keywords:** remote sensing image interpretation, dynamic land use change, Markov–ANN–CA model, driving force, simulation-based prediction

## Abstract

Until now, few studies have used the mainstreaming models to simulate the land use changes in the cities of rapid urbanizing regions. Therefore, we aimed to develop a methodology to simulate the land use changes in rapid urbanizing regions that could reveal the land use change trend in the cities of the regions. Taking the urban areas of Wuhan, a typical rapid urbanizing region in China, as the study area, this study built a Markov chain–artificial neural network (ANN)–cellular automaton (CA) coupled model. The model used land use classification spatial data with a spatial resolution of 5 m in 2010 and 2020, obtained by remote sensing image interpretation, and data on natural and socio-economic driving forces for land use change simulation. Using the coupled model, the land use patterns of Wuhan urban areas in 2020 were simulated, which were validated in comparison with the actual land use data in 2020. Finally, the model was used to simulate the land uses in the study area in 2030. The model validation indicates that the land use change simulation has a high accuracy of 90.7% and a high kappa coefficient of 0.87. The simulated land uses of the urban areas of Wuhan show that artificial surfaces will continue to expand, with an area increase of approximately 7% from 2020 to 2030. Moreover, the area of urban green spaces will also increase by approximately 7%, while that of water bodies, grassland, cropland, and forests will decrease by 12.6%, 13.6%, 34.9%, and 1.3%, respectively, from 2020 to 2030. This study provides a method of simulating the land use changes in the cities of rapid urbanizing regions and helps to reveal the patterns and driving mechanisms of land use change in Wuhan urban areas.

## 1. Introduction

Rapid urbanization has significantly changed the way in which humans use land. It has become a great concern worldwide because it leads to biodiversity decline, ecosystem degradation, land resource loss, environmental pollution, and other problems [1,2,3]. In 1993, the International Human Dimensions Programme on Global Environmental Change and the International Geosphere–Biosphere Programme jointly launched the Land-Use and Land-Cover Change project [4]. In the early 2000s, the Global Land Project was started, focusing on the integration and the simulation study of land as a coupled human–environment system. The dynamic monitoring of land use and land use simulation based on this conceptual coupled human–environment system has since received increasing interest from researchers [5].

Since the reform and opening-up of China in 1978, socio-economic development and industrialization have increased the country’s urban population. Moreover, rural lands around cities have been turned into urban lands, leading to the rapid expansion of urban and suburban areas [6,7]. Since the 1990s, an increasing number of researchers have studied the land use changes from both the national [8] and regional scales [9,10,11] and built models based on land use driving forces to explain the course of land use changes and forecast their trends. Most of these studies simulated land use in terms of area and spatial distributions by using quantitative, spatial, and coupled quantitative–spatial models. Quantitative models, such as the system dynamics [12], regression [13], logistic [14], and gray relational analysis models [15], work on mathematical reasoning frameworks and focus on the quantitative changes of land use types. Spatial models, such as the cellular automaton (CA) [16], land change modeler (LCM) [17], conversion of land use and its effects (CLUE-S) [18], and future land use simulation (FLUS) [19] models, reveal land use changes and their correlations with driving forces at different spatial and temporal scales with the help of the remote sensing and Geographic Information System technologies. However, given the unsatisfactory simulation accuracy of the abovementioned models, spatial models must be used together with quantitative models to make reasonable simulation-based predictions of land use [9,20,21].

The models demonstrated in the preceding paragraph cannot well reflect the internal mechanisms of land use changes, as the spatial patterns and changes of land use involve highly complex and nonlinear processes; hence, accurate land use simulation using general mathematical formulae and models is difficult. To increase the accuracy of land use simulation, the characteristics of the spatial and temporal changes of land use and the driving forces are required. Therefore, quantitative and spatial models have been coupled to predict the changes of land use patterns over time with a higher accuracy [22,23,24]. Al-sharif and Pradhan [25] simulated the urban land use change of Libya from 1984 to 2010 based on the CA–Markov model. Gharaibeh et al. [26] coupled artificial neural network (ANN), CA, and Markov models to simulate the land use change of Irbid in 2015 and compared it with the land use change simulated by the CA–Markov model. The results showed that the ANN–CA–Markov model’s simulation better reflected the real changes in the land use patterns. Meanwhile, Zhang et al. [27] built an ANN–CA–Markov model based on the nighttime light data from 1992 to 2017 and simulated the changes of urban built-up areas in China in 2017. The kappa coefficient of the simulation results was 0.78, indicating that the model was suitable for predicting the expansion of cities. Owing to its non-aftereffect feature, the Markov model that was used in the previous studies predicts the change of land use types over time based on the transition probability matrix [28]. The CA model obtains land use transition rules based on the neighborhood and land use suitability [29]. The ANN model captures the nonlinear relations between each land use type and driving forces and deals with complex systems, such as urban growth and land use changes [30]. In other words, the ANN, CA, and Markov models can be coupled to accurately simulate the changes of the land use patterns in a city. However, few studies use the mainstreaming models to simulate the land use changes in the cities of rapid urbanizing regions (A rapid urbanizing region is a region in the process of rapid urbanization, which includes urban areas and suburban/rural areas that are transformed into urban areas within a relatively short period.) because of the highly spatial heterogeneity, high temporal dynamics, and complex driving forces of the land uses in the cities [16,30].

Given the knowledge gap demonstrated above, this study aimed to develop a methodology to simulate the land use changes in rapid urbanizing regions that could reveal the land use change trend in the cities of the regions. To achieve the objective of the study, we built a land use change model coupling ANN, Markov chain, and CA to simulate and predict the spatial land use changes of Wuhan urban areas, a typical rapid urbanizing region, based on the 2010 and 2020 land use classification data that were obtained by remote sensing image interpretation, considering the natural, socio-economic, and other driving forces. This study provides a method of simulating the land use changes in the cities of rapid urbanizing regions and helps to reveal the patterns and driving mechanisms of land use change in Wuhan urban areas.

## 2. Study Area

**Figure 1:**
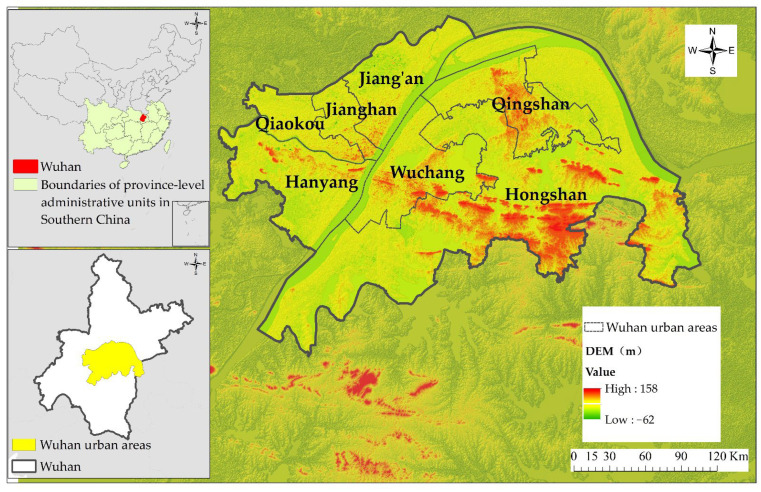
Location of the study area.

The urban areas of Wuhan (Figure 1), including the Jiangan, Jianghan, Qiaokou, Hanyang, Wuchang, Hongshan, and Qingshan districts, were chosen as the study area. Wuhan urban areas are the central region in central China and are dominated by low hills and plains that are mainly covered by fluvo-aquic and paddy soil, with altitudes ranging from −62 to 158 m. The monsoon-influenced humid subtropical climate brings an average annual precipitation of 1150 to 1450 mm and maintains an average annual temperature of 15.8 to 17.5 °C in the region. Located at the confluence of the Yangtze and Hanjiang rivers, this region boasts abundant water resources. It has 38 lakes and reservoirs that supply an average annual water resource of 539 million m^3^. By the end of 2019, 6.4021 million residents lived in the urban areas of Wuhan, leading to a local GDP of CNY 784.373B, which is 2.4 times that in 2010, with the output of primary industry up from CNY 317M to CNY 966M. The output of secondary and tertiary industries increased by CNY 12.728B and CNY 285.5B, respectively, by the end of 2019. Wuhan urban areas experienced a rapid urbanization process with a population increase from 6.1 million to 7.2 million (an increase of 18.4%) from 2010 to 2020.

## 3. Methodology

### 3.1. Data Sources

To explore the pattern of land use changes within the city at a finer resolution, 5 m resolution remote sensing images produced by the RapidEye satellite in 2010 and 3 m resolution remote sensing images produced by the SuperDoves satellite in 2020 were used and interpreted to generate the land use classification data. The geographic data mainly included the elevation, slope, and aspect data. The Advanced Land Observing Satellite Digital Elevation Model data with a 12.5 m resolution were obtained from the NASA website (https://search.asf.alaska.edu/#/ (accessed on 18 May 2022)). The Slope and Aspect tools in ArcGIS10.7 were used to obtain the spatial raster data of the slope and the aspect of the urban areas of Wuhan. The population density data were mainly obtained from WorldPop (https://www.worldpop.org/ (accessed on 18 May 2022)) with a 100 m resolution. The vector data of the urban areas were converted from Google Earth, and those of the rivers and roads were extracted from remote sensing images. A total of 72,455 points of interest, including financial institutions, scenic spots, shopping stores, restaurants, medical institutions, and recreational facilities, were obtained by a Python crawler from Amap. The data on the distance to urban centers, rivers, roads, financial institutions, scenic spots, shopping stores, restaurants, medical institutions, and recreational facilities were generated using the Euclidean Distance tool of ArcGIS10.7.

To avoid spatial mismatch between different data, the Universal Transverse Mercator projection was used for all vector and raster data in ArcGIS10.7 based on the WGS_1984_UTM_Zone_50N coordinate system. The data were resampled to a 5 m spatial resolution.

### 3.2. Land Use Classification and Accuracy Validation

Previous studies showed that supervised classification produced better results than unsupervised classification [31,32]. Therefore, a land use classification system for the urban areas of Wuhan (Table 1) was established according to the *Land Use Classification Standard* (GB/T 21010–2017). The preliminary land use classification map was produced by using the remote sensing images that were pre-processed by ENVI5.3, based on the maximum likelihood method in supervised classification. The land use classification was modified by visual interpretation to improve the classification accuracy. In this work, land use is classified into the six types: artificial surface (AS); water body (WB); cropland (CL); forest (F); grassland (GL); and urban green space (UGS). Distinguishing urban green spaces from forests and grasslands was difficult; thus, we first classified artificial surfaces, water bodies, croplands, forests, and grasslands, and then identified the urban green spaces using the boundary of Wuhan city. To keep the data resolution consistent, the 3 m resolution land use classification data of 2020 were resampled to a 5 m resolution using the Majority resampling type in the resampling tool of ArcGIS10.7.

The confusion matrix and the kappa coefficient were used to examine the data reliability of the classification results [33]. Using Google Earth, 1083 and 1045 points were randomly and evenly selected from the remote sensing images that were acquired in 2010 and 2020, respectively, to generate the validation samples of the six land use types. These points were then used to build confusion matrices and obtain the user, producer, and overall accuracies and the kappa coefficients of each land use type.

The overall accuracy refers to the ratio of the number of correctly classified pixels to the total number of pixels and is calculated as follows:

(1) $$OA=\frac{\sum_{i=1}^{n}X_{ii}}{N}$$

The user accuracy refers to the ratio of the number of correctly classified pixels to the total number of classified pixels of a specific land use type and is calculated as follows:

(2) $$U_{a}=\frac{X_{ii}}{X_{+i}}$$

The producer accuracy refers to the ratio of the number of correctly classified pixels to the total number of actual pixels of a specific land use type and is calculated as follows:

(3) $$Pa=\frac{X_{ii}}{X_{i+}}$$

The kappa coefficient can objectively describe the classification accuracy of each land use type and is calculated as follows:

(4) $$K=\frac{N\sum_{i=1}^{n}X_{ii}-}{N^{2}-\sum_{i=1}^{n}\left(X_{i+}\times X_{+i}\right)}$$

where: *K* is the kappa coefficient; *n* is the number of land use types; *N* is the total number of pixels; *X_ii_* represents the number of correctly classified pixels; and *X_i+_* and *X_+i_* represent the total number of actual pixels of land use type *i* and the total number of classified pixels of land use type *i*, respectively. The kappa value ranges between [0, 1]. It indicates inconsistency between the classified and actual land use when the kappa value is between 0 and 0.2, general consistency when it is between 0.21 and 0.4, moderate consistency when it is between 0.41 and 0.6, high consistency when it is between 0.6 and 0.8, and full consistency when it is between 0.81 and 1.0.

### 3.3. Simulation of Land Use Change

#### 3.3.1. Correlation and Collinearity Analysis of Influencing Factors

The process of land use change is highly complex and influenced not only by natural conditions, but also by socio-economic factors and geographic locations; thus, the selection of driving forces should consider data characteristics, such as accessibility, consistency, comprehensiveness, and quantifiability [34,35,36].

The following driving forces were selected in this study: slope, aspect, elevation, population density, and the distances to urban centers, rivers, roads, financial institutions, scenic spots, shopping stores, restaurants, medical institutions, and recreational facilities. To avoid the influence of correlations and collinearity among the driving forces on the model, 1,000,000 samples were randomly selected from the 13 types of driving forces using MATLAB 2017b. The Pearson correlation coefficient (r) and the variance inflation factor (VIF) were calculated using SPSS.

The correlation matrices showed that the distance to shopping stores and financial institutions and that to recreational facilities and restaurants were highly correlated, with the correlation coefficients greater than 0.8. The distance to medical institutions and that to financial institutions and the distance to shopping stores and that to restaurants were moderately correlated, with the correlation coefficients higher than 0.7. The distance to restaurants showed a strong collinearity with that to recreational facilities because the multicollinearity results indicated that the variance inflation factors of these two were greater than 10.

In summary, the following nine factors were selected as input for the logistic and Markov–ANN–CA models: distance to urban centers, rivers, roads, scenic spots, and financial institutions; population density; slope; elevation; and aspect.

#### 3.3.2. Logistic Regression Analysis

A logistic regression analysis calculates the relation between a dependent variable and multiple independent variables to explain the impact and effects of each variable on event occurrence [37]. In this study, binary logistic regression was used to quantitatively calculate the odd ratio of the driving forces, according to the following formula:

(5) $$\log\left(\frac{P_{i}}{1-P_{i}}\right)=\beta_{0}+\beta_{1}X_{1,i}+\beta_{2}X_{2,i}+\cdots +\beta_{n}X_{n,i}$$

where $X_{1,i}$–$X_{n,i}$ are the driving forces and $\beta_{0}$–$\beta_{n}$ are the regression coefficients of the respective driving forces. If *β* > 0, the driving factor is positively correlated with the corresponding land use type; otherwise, they are negatively correlated.

The regression coefficient *β* cannot directly present the odd ratio; hence, Exp(*β*) is introduced to replace *β*. If Exp(*β*) > 1, the proportion of the land type increases; otherwise, it decreases [38]. To determine the correlation degree between each land use type and the driving forces, the aforementioned nine driving forces were converted to the ASCII format in ArcGIS10.7, and the presented data were loaded into MATLAB 2017b. One million data records were randomly sampled from the nine driving forces and exported to an Excel file, which was used to extract the binary data of the six land use types. A regression analysis was then performed in SPSS with the nine driving forces as the independent variables and the six land use types as the dependent variables to obtain the odd ratio for each driving force for different land use types (Table 2). In the table, X_1_–X_9_ represent the distance to urban centers, water bodies, roads, financial institutions, and scenic spots, population density, slope, elevation, and aspect, respectively.

#### 3.3.3. Land Use Suitability Analysis

The spatial development strategy for land use in Wuhan indicates that maintaining economic growth, protecting resources, and balancing land use and ecological development are important ways of protecting the ecosystem and making good use of the restrictive landscape of Wuhan. Priority must be given to ecological land planning to prevent the irrational expansion of the urban area. Accordingly, the land use simulation of the urban areas of Wuhan requires an evaluation of land use suitability based on natural and socio-economic factors. The obtained suitability of the croplands, forests, and grasslands should be used as the constraint factors in the Markov–ANN–CA model to ensure that the simulation results are consistent with the spatial pattern of the urban areas.

(1) Selection of the evaluation factors

Land use suitability is the result of a comprehensive evaluation of several factors that are closely related to the land use types. Six factors were selected considering the odd ratio that was obtained in the logistic regression: distance to roads and water bodies, elevation, slope, aspect, and land use.

(2) Ranking and weight of the evaluation factors

Calculating the land use suitability in the case of a large number of evaluation factors and inconsistent attributes is difficult. Therefore, the evaluation factors for each land use type must be graded to keep the factor values within certain ranges. According to previous studies [39,40,41] and considering the topographic and geomorphological characteristics of the urban area of Wuhan, the evaluation factors were graded into five classes, with values 5, 4, 3, 2, and 1 representing the degree of suitability. The larger the value, the higher the degree of suitability. Table 3, Table 4 and Table 5 are the grading standards for the metrics of croplands, forests, and grasslands.

Evaluation factors have been weighted according to the objects, purposes, and contents of the evaluation by using various models in previous studies, such as the analytic hierarchy process (AHP) [42], logistic regression [43], fuzzy comprehensive evaluation [44], and multi-criteria decision analysis [45]. In this study, the weight of each factor was obtained by AHP based on yahp10.3. First, the evaluation hierarchy was established according to the study area conditions. Second, the judgment matrix between the factors was built according to the logistic regression results. Finally, a consistency check was performed to verify the judgment matrix suitability. When building the judgment matrix, the relative importance of any two factors was determined according to their odd ratio. The weights of the evaluation factors of the suitability of cropland, forest, and grassland are provided in Table 6.

(3) Comprehensive evaluation of land use suitability

The comprehensive evaluation of suitability is a comprehensive analysis of evaluation factors. Each evaluation factor of each land use type is multiplied by its weight and added together to obtain the final evaluation result of the land use suitability. The calculation formula for land use suitability is as follows:

(6) $$R_{i=}\sum_{j}^{n}W_{j}*A_{ij}$$

where $R_{i}$ means the land use suitability of the *i*th cell, $A_{ij}$ indicates the normalized value of the jth suitability evaluation factor of the ith cell after grading, and $W_{j}$ is the weight of the jth evaluation factor.

#### 3.3.4. Markov–ANN–CA Model

A Markov model can simulate the number of future land use types based on the current types and trends, but it cannot simulate the spatial changes of land use [46]. A CA model can simulate complex temporal and spatial changing processes through a powerful spatial computation; however, its transition rules and model structure are difficult to determine [47,48]. An ANN model is suitable for simulating complex nonlinear systems and allows synthetic data training, thereby obtaining a high simulation accuracy [49,50,51]. Combining the three models can give full play to their respective advantages.

The open and dynamic characteristics of urban systems make the relation between urban land use types complex. Therefore, in the land use simulation process, the factors affecting the land use must be considered, and the mapping relation between them and the land use types must be determined. By combining the ANN model and training the neural network, the CA rules and related parameters can be obtained to improve the simulation accuracy and provide a reference basis for urban planning. In this study, MATLAB 2017b was used to integrate the CA, ANN, and Markov models. With the aid of the ANN and Markov models, the shortcomings of the traditional CA model were eliminated, making it possible to simulate and predict land use in the future based on the actual land use changes in the urban areas of Wuhan.

In the modeling process, the tool IDRISI for the Markov model was first used to predict the land use demand in 2030 in the urban areas of Wuhan. This land use demand was further used as the condition to stop the iteration in the land use simulation program. For the prediction, the land use classification data of 2010 and 2020 were loaded into IDRISI software and saved in .rst format in the reclassification module before being input into the Markov tool. Setting the time interval to 10, the transition probability matrix of 2010–2020 was obtained on the basis of 2010 and used for 2030.

The ANN model was then trained with nine driving forces, three constraint factors, the neighbor data of six types of land use, and land use data of 2010 to obtain the transition rules of the CA by using the CA model. In the process of the training of the ANN model and the simulation of the land use in 2020, the CA transition rules were obtained by creating a back-propagation three-layer feed-forward neural network to train the relations between each driving factor and land use types. The neighborhood of a cell is an important factor that affects its change; hence, the neighbor factor of the land use type is also used as a driving factor of the Markov–ANN–CA model. Given the abovementioned driving forces, a 19–16–6 structure is defined for the neural network—that is, 19 driving forces in the input layer, 16 hidden layers, and the transition probability of the six land use types in the output layer. The training function, excitation function for the hidden layer, and excitation function for the output layer are the Levenberg–Marquardt (LM) algorithm, tansig, and logsig, respectively. A total of 300,000 raster cells were randomly selected as the training data from the 2010 land use data of the study area using MATLAB 2017b. When the neural network was trained for 500 iterations, its root mean square error was 0.011084, meeting the accuracy requirement and indicating that the neural network can be used to simulate the land use of the study area in 2020.

The 2010 land use data of the urban areas of Wuhan was input to the trained neural network to calculate the probability of each cell transiting into one of the six land use types. A random variable was introduced to perturb the transition probability, which was then compared with the threshold value to obtain the specific land use type. Subsequently, this process was repeated for the determined land use types until the difference between the quantity of a certain type and that predicted by the Markov model was within 30,000. The simulated land use data of the urban areas of Wuhan in 2020 were then generated.

The driving forces that were selected by the ANN–CA model were associated with the *n* attributes of each simulated cell. These attributes collectively determined the probability of the land use transition for each cell at the time *t*, expressed by the following formula:

(7) $$X\left(k,t\right)={\left[x_{1}\left(k,t\right),x_{2}\left(k,t\right),x_{3}\left(k,t\right),\cdots ,x_{i}\left(k,t\right),\cdots x_{n}\left(k,t\right)\right]}^{T}$$

where $x_{i}\left(k,t\right)$ is the ith variable of cell k at the time t.

To avoid the training errors caused by a numerical inconsistency, the data input to the ANN model was normalized with the maximum and minimum values before being input to the hidden layer. The calculation formulae are

(8) $$x_{i}\left(k,t\right)=\left[x_{i}\left(k,t\right)-min\right]∕\left(\max-min\right)$$

(9) $$net_{j}\left(k,t\right)=\sum_{j}w_{i,j}x_{i}^{\prime }\left(k,t\right)$$

where $net_{j}\left(k,t\right)$ is the value that is received by the jth neuron of the hidden layer from the input layer, and $w_{i,j}$ is the weight of the input and hidden layers.

The signal from the hidden layer to the output layer is the transition probability, which is calculated as follows:

(10) $$P\left(k,t,l\right)=\sum_{j}\;w_{j,l}\frac{1}{1+e^{-net_{j}\left(k,t\right)}}$$

where P(k,t,l) denote the probability of the land use transition of cell k from the present type to type l at the simulation time t, and $w_{j,l}$ is the weight of the hidden layer to the output layer.

To make the simulation results more accurate, a random variable is introduced to the CA model with the following expression:

(11) $$RA=1+{\left(-\ln\gamma \right)}^{\alpha }$$

where γ is the random number falling in the range of [0, 1], and α is the parameter controlling the random variable value.

Given the abovementioned conditions, the probability of the land use transition of cell *k* from the present type to type *l* at the simulation time *t* is:

(12) $$P\left(k,t,l\right)=\left[1+{\left(-\ln\gamma \right)}^{\alpha }\right]\times \sum_{j}w_{j,l}\frac{1}{1+e^{-net_{j}\left(k,t\right)}}$$

The transition probability of each land use type was obtained in each neural network operation cycle. The magnitude of its value represented the transition probability from the current land use type to the other land use types. The larger the value, the higher the transition probability. The values ranged from 0 to 1 during the whole operation. The values of the land use transition probability were small in a short period of time. Several simulations were needed to determine the direction of the land use transition. Therefore, the threshold value T and the random variable *α* were used as the parameters for controlling the land use transition. The calculated transition probability was compared with the threshold value. The land use type of the cell was changed when the transition probability of land use was greater than the threshold value. A random number was generated when the transition probability of land use was less than the threshold value. When the random number was greater than 0.5, the land use type corresponding to the maximum transition probability value was output. No transition occurred when the random number was less than 0.5. To verify the accuracy criteria, the Lee–Sallee shape index was used based on the following formula [52]:

(13) $$L=\frac{A_{0}\cap A_{1}}{A_{0}\cup A_{1}}$$

where $A_{0}$ is the land use classification of the actual year and $A_{1}$ is the land use classification of the simulated year. The simulation result was good when the value of the Lee–Sallee index was between 0.3 and 0.7.

When further exploring the mechanism of the land use changes in the urban areas of Wuhan, the factors with strong correlations and collinearity were first removed by correlation and collinearity analyses. Three constraint factors, namely the suitability of croplands, forests, and grasslands, were obtained by using logistic regression and a suitability analysis to control the disorderly expansion of each land use type. Finally, the coupled Markov–ANN–CA model was built to simulate the land use in 2020 based on the 2010 data. The model was validated by using the actual land use data in 2020 and used to simulate the land use in 2030 in the urban areas of Wuhan. Based on the simulation, we analyzed the land use changes from 2020 to 2030.

## 4. Results

### 4.1. Land Use Classification Results and Accuracy Evaluation

Table 7 shows that the overall accuracy in 2010 was 0.91, and the kappa coefficient was 0.88. The overall accuracy in 2020 was 0.93, and the kappa coefficient was 0.91. Given the high accuracy of land use classification in 2010 and 2020, the land use data can be used to predict the land use in the urban areas of Wuhan in 2030.

### 4.2. Land Use Suitability

**Figure 2:**
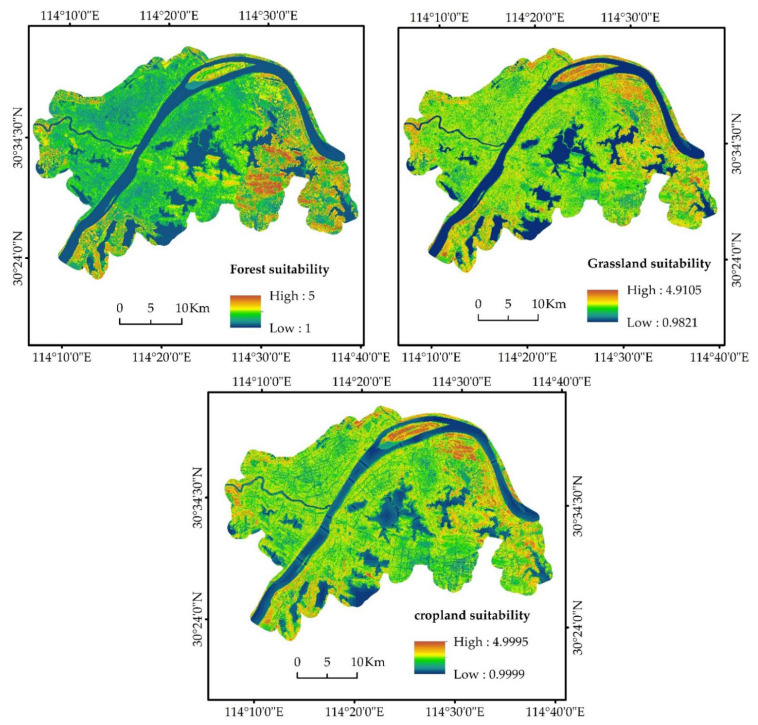
Spatial patters of the suitability of cropland, grassland, and forest in Wuhan urban areas in 2020.

Using the weighted sum tool of ArcGIS10.7, the normalized value of the evaluation factors of the suitability of croplands, forests, and grasslands were multiplied by their weights to obtain the suitability of cropland, grassland, and forests in 2020 (Figure 2).

### 4.3. Simulation-Based Prediction of Land Use Change

#### 4.3.1. Predicted Land Use Quantity Using the Markov Model

Using the Markov model, the land use demand of 2020 and 2030 were obtained through a matrix operation (Table 8).

**Figure 3:**
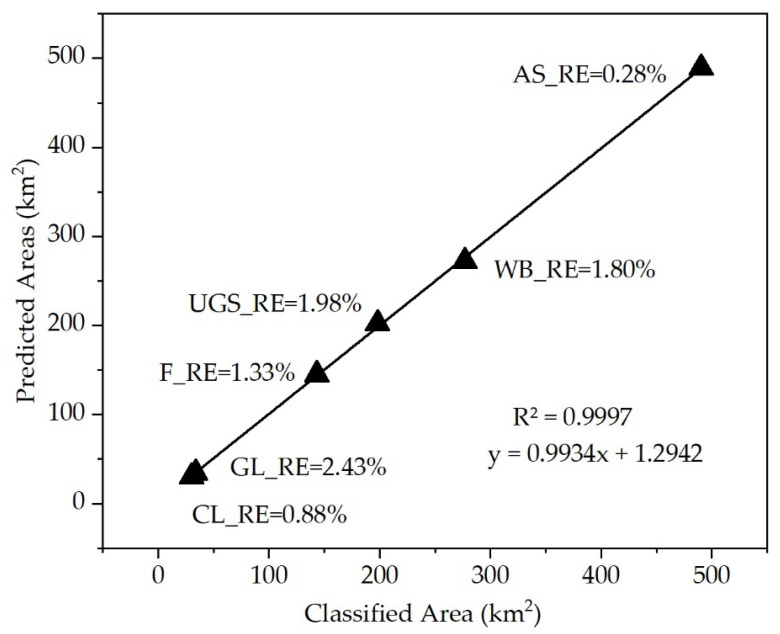
The error of Markov chain in 2010. (RE is the relative error between the simulated area of each land use and the actual classification area).

To evaluate the prediction performance of the Markov model, a scatter diagram was generated with the actual land use area in 2020 and the predicted land use area to compare the difference between the two. Figure 3 shows that R^2^ was higher than 0.99, and the predicted land use area that was obtained using the transition probability matrix was highly consistent with the actual area of the classified land use types. The relative error between the two was between 0.28% and 2.43%, indicating that the Markov model exhibited a satisfactory prediction performance. The prediction results can be used in the CA model to stop iteration.

#### 4.3.2. Simulation of Land Use in 2020

Different combinations of the random variable a and the transition threshold T can affect the simulation accuracy of the land use in the urban areas of Wuhan. Therefore, the best parameters can be obtained by setting different combinations. Table 9 shows the accuracy of the land use simulation with different combinations. The results of several tests indicated that the model simulation had the highest accuracy when a = 2 and T = 0.8.

#### 4.3.3. Consistency Check between the Simulated and Actual Land Use

**Figure 4:**
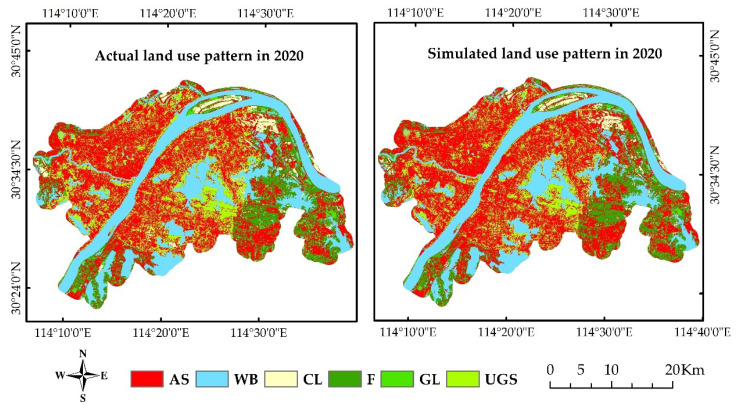
Actual (left) and simulated (right) land use patterns in Wuhan urban areas in 2020.

The land use simulation of the urban areas of Wuhan in 2020 that was obtained from the abovementioned model simulation is shown in Figure 4. The quantitative accuracy check and the confusion matrix were adopted to examine the consistency between the actual and simulated land uses in 2020 and the accuracy of the land use simulation with the random variable a = 2 and the threshold T = 0.8. In Table 10, the error between the simulated and actual cell number of the land use type in 2020 was between 0.7% and 7.39%, and the simulation accuracy was 90.97% with a kappa coefficient of 0.87. These results indicate that the simulation accuracy of the 2020 land use in the study area was high, and the model had a high confidence level.

#### 4.3.4. Spatial Simulation and Land Use Changes

If the natural and socio-economic factors and the land use policies in the urban areas of Wuhan do not significantly change from 2020 to 2030, the land use changes in 2030 can be simulated based on the land use transition rules from 2010 to 2020. Using the land use data in 2020, the Markov–ANN–CA model was used to simulate the land use in 2030 in the urban areas of Wuhan based on the trained neural network, with the random variable set to 2 and the threshold value set to 0.8.

**Figure 5:**
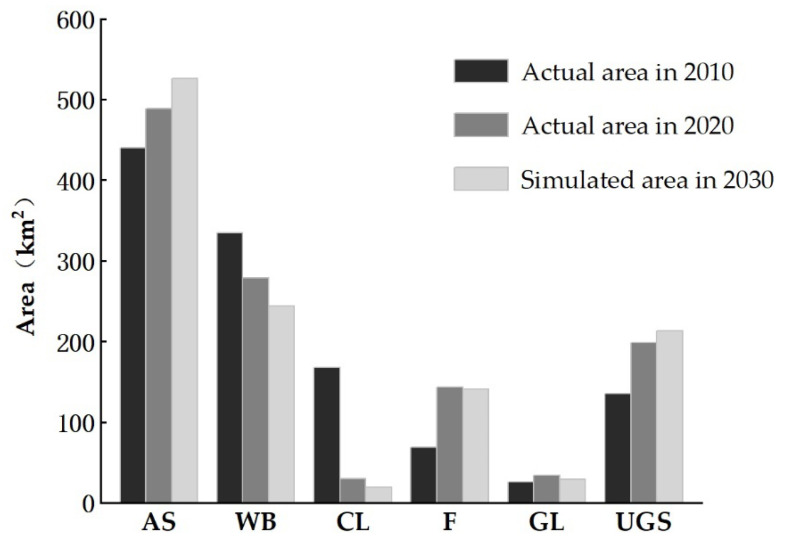
Area change of different land use types in Wuhan urban areas from 2010 to 2030.

For a quantitative analysis of the land use changes from 2010 to 2030, the actual cell number and area of land use types in 2010 and 2020, and the simulated cell number and area of land use types in 2030 are listed in Table 11 and Figure 5. The results show that the area of artificial surfaces and urban green spaces in the urban areas of Wuhan continue to increase from 2010 to 2030; the area of water bodies and croplands continue to decrease; and the area of forests and grasslands are almost unchanged. Comparing the trends of the land use change from 2010 to 2030, the increase in the area of artificial surfaces and urban green spaces from 2020 to 2030 is smaller than that from 2010 to 2020. The decrease in the area of water bodies and croplands from 2020 to 2030 is smaller than that from 2010 to 2020. From 2020 to 2030, the area of artificial surfaces and urban green spaces will increase by approximately 7%, while those of water bodies, grasslands, croplands, and forests will decrease by 12.6%, 13.6%, 34.9%, and 1.3%, respectively. These changes imply that the predicted trends of the land use change are consistent with the environmental protection policies of Wuhan.

**Figure 6:**
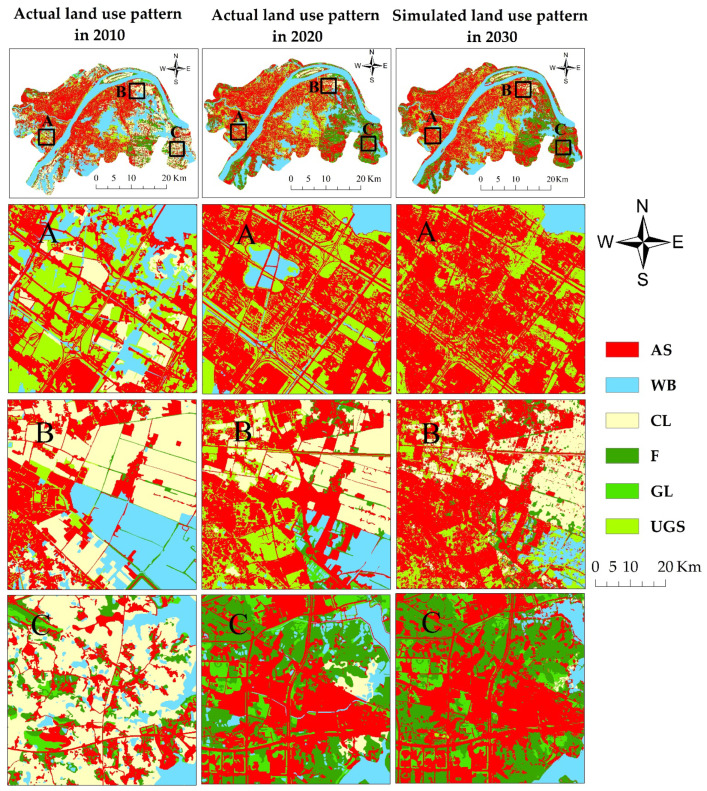
The spatial patterns of land use in Wuhan urban areas in 2010 (actual), 2020 (actual), and 2030 (simulated). The maps numbered (**A**–**C**) show zoomed in areas in the western, northern, and eastern parts of the study area, respectively.

To explore the changes in land use in the different areas of Wuhan’s urban areas from 2010 to 2030, three areas were selected for analysis in the western (A), northern (B), and eastern (C) parts of the study area (Figure 6). The artificial surfaces expand and become more aggregated in all three parts. The urban green spaces become scattered and are mostly distributed continuously in a linear pattern on both sides of urban roads due to the expansion of the artificial surfaces. Consequently, this reduces the area of urban green spaces. The area of croplands maintains a decreasing trend in the three parts. The croplands outside the urban areas become more and more scattered, mainly due to economic development and the conversion of croplands to artificial surfaces and forests. Forests are mainly distributed in the urban periphery. From 2010 to 2020, forests changed from a sporadic to an aggregated distribution due to the influence of various factors, including the policy of converting cropland to forests and the construction of a sightseeing and leisure forestry belt in the Hongshan District. The area of forests largely changed from 2010 to 2020 but will not change much from 2020 to 2030. The area of water bodies in the western and northern parts of the study area maintained a decreasing trend from 2010 to 2030. This may be because of the development of secondary and tertiary industries and the increasing demand for artificial surfaces, which occupies the land that was originally used for fishery and aquaculture.

## 5. Discussion

This study used a coupled Markov-CNN-CA model to simulate the land use changes in Wuhan urban areas. The comparison of our method to existing methods used in similar study areas shows that our method can generate more accurate land use change simulation in rapid urbanizing areas with a kappa coefficient of 0.88 and an overall accuracy of over 0.90. For example, one land use simulation using a Markov-CA model in the rapid urbanizing areas in Central Iran has a Kappa coefficient of 0.82 [53]. Another two examples are the land use change simulation in the whole of Wuhan using the FLUS model, which has an overall accuracy of 0.75 [54], and the land use simulation using the LCM model in the Greater Accra Metropolitan Area, which has a kappa coefficient of 0.76 [55]. Moreover, our simulation can reflect the land use changes inside the Wuhan city with a relatively high spatial resolution. In comparison, previous land use simulations using the same method as ours, such as the one in the Irbid city, Jordan [26] and the one in the urban areas of China [27] only show the future urban sprawl directions, but do not reveal the land use change characteristics inside the cites. Despite the advantages, there are some limitations in our land use simulation method. One major limitation is that we did not consider the soil physical and chemical properties and the geological conditions in selecting the indicators of land use suitability because of the lack of localized spatial data. Another major limitation is that we did not consider the policy factors in selecting the driving forces of land use changes because of the lack of spatial data on the local land use policies. These two limitations may reduce the accuracy of our land use simulation and should be overcome in future studies. 

We found that there were large increases in the area of artificial surfaces and urban green space and a large decrease in the area of cropland in Wuhan urban areas from 2010 to 2020 (Figure 5). These findings are consistent with the studies of Sun et al. [56] and Wu et al. [57]. The driving mechanism of these land use change trends was the fast urbanization of the central urban areas of Wuhan from the 1990s and the vigorous promotion of the integrated development of the urban and rural areas in Wuhan in 2000 [4]. These reasons led to the successive development of various industrial parks and real estate. Moreover, the land use simulation shows that the increase in the area of artificial surfaces and urban green spaces in 2020–2030 will be smaller than that in 2010–2020 and the decrease in the area of cropland in 2020–2030 will be much smaller (Figure 5). The possible driving force for this land use change trend is that Wuhan initiated a new urbanization process plan in 2014, during which the city pattern already formed, and the urban expansion will slow down. Anther noticeable finding is that the increased artificial surfaces and urban green space in 2020–2030 will be more spatially fragmented than those in 2010–2020 (Figure 6). The possible reason for this phenomenon is that many of the cropland patches that can be occupied by the expansion of impervious surfaces and urban green space will be small and scattered ones in the period of 2020–2030. 

Wuhan urban areas have a coupled human–environmental system, as different types of ecosystems are closely interconnected and human activities and environmental processes highly interact [57] Therefore, it is necessary to rationally implement land use planning and management in order to promote the harmonious coexistence between the human system and the natural environmental system. To optimize the land use in Wuhan urban areas, we propose recommendations for future land use from three perspectives. First, our study showed that most of the increased impervious surfaces came from the occupied cropland in Wuhan urban areas. Therefore, we recommend that the Wuhan Prefecture government build a system to conserve basic farmland, increase the funds for cropland conservation, and build a system to supervise the farmer’s homesteads. Second, as there are many hills and water bodies in Wuhan urban areas, the hills and water bodies can be used to optimize the land use structure. For example, the unused land on the hills can be used to build parks and some water bodies along the Yangtze River and the Hanjiang River can be used to create wetland parks. These newly built parks will not only increase the ecosystem quality but also benefit the mental health of the urban residents. Third, as shown by our study that the water bodies exhibit a shrinking trend in 2010–2030 in Wuhan urban areas, it is necessary to implementing conservation and restoration measurements for water bodies. Possible measurements include connecting rivers and lakes, avoiding converting water bodies to constructed land, and desilting rivers.

## 6. Conclusions

This study aimed to develop a methodology to simulate the land use changes in rapid urbanizing regions that can reveal the land use change trend in the cities of the regions. We used Wuhan urban areas, a typical rapid urbanizing region in Central China, as the case area, obtained classified land use data with a high spatial resolution by remote sensing images interpretation, simulated the land use with a Markov–ANN–CA coupled model, and analyzed the land use changing trends. The results show that: (1) The land use classification accuracy is high with Kappa coefficients over 0.88. (2) The comparison between the simulated land use data and the actual land use data in 2020 indicates a high simulation accuracy with a 0.87 kappa coefficient. (3) The land use simulation in 2030 shows that the increase in the area of artificial surfaces and urban green spaces, and the decrease in the area of water bodies and cropland will become smaller than those in 2010–2020. (4) The increased artificial surfaces and urban green spaces in 2020–2030 will be more spatially fragmented than those in 2010–2020.

The results of our study indicate that the Markov–ANN–CA coupled model exhibits a satisfactory performance in the land use simulation of rapid urbanizing regions and can help to reveal the land use change trend in the cities. Moreover, our findings show that the urbanizing process will slow down in 2020–2030, compared to that in 2010–2020 in Wuhan urban areas. Based on the results, we propose recommendations for future land use to promote the harmonious coexistence of the coupled human–environmental system in Wuhan urban areas from the perspectives of cropland conservation, land use structure optimization, and avoiding water body shrinking. Future research may consider land use plans and policies as the driving force factors in land use change simulation to improve the model performance.

## Figures and Tables

**Table 1:** Land use classification of Wuhan urban areas.

Land Use Class	Description
Artificial surface (AS)	Surfaces where landscape has been changed by human construction activities by replacing natural surfaces (urban land, rural residential land, and roads in this study)
Water body (WB)	Rivers, lakes, ponds, and reservoirs
Cropland (CL)	Dry land and paddy fields
Forest (F)	Natural forest and artificial forest
Grassland (GL)	Natural grassland and artificial grassland
Urban green space (UGS)	The grassland and forest within the boundary of Wuhan city

**Table 2:** Odd ratio for each driving forces for different land use types.

	Land Use Classes	AS	WB	CL	F	GL	UGS
Driving Forces							
X_1_		0.999968	0.999987	1.000012	1.000099	1.000192	0.999988
X_2_		1.000249	0.99935	1.000301	1.000101	1.000035	0.999925
X_3_		0.999316	1.000525	1.000141	0.999668	0.999265	0.999878
X_4_		0.999656	1.000144	1.000007	1.000005	0.999991	0.999646
X_5_		0.999977	0.99999	1.000132	1.000049	1.000058	0.999702
X_6_		1.004947	0.990147	0.979198	0.969826	0.980787	0.998772
X_7_		0.978571	1.016375	0.964949	1.029236	1.009155	1.024573
X_8_		1.019248	0.733384	0.997132	1.054774	1.023229	1.026238
X_9_		1.001458	0.998009	1.002076	1.000462	1.000909	1.000658
Constant		0.910857	6.285594	0.076477	0.019841	0.01033	0.288609

**Table 3:** Grading of the suitability evaluation indicators for cropland.

Indicators	5	4	3	2	1
Distance to road	>2000	1500–3000	1000–1500	100–1000	<100
Distance to river	<1000	1000–2000	2000–4000	4000–6000	>6000
DEM	6–15	15–20	20–30	30–40	<6 & >40
Slope	4–6	6–12	12–16	16–20	<4 & >20
Land use	CL	GL	F	WB	AS&UGS
Aspect	south, Fat	southeast	east, southwest	north, northwest	west, northeast

**Table 4:** Grading of the suitability evaluation indicators for forest.

Indicators	5	4	3	2	1
Distance to river	<1000	1000–2000	2000–4000	4000–6000	>6000
DEM	6–15	15–20	20–30	30–40	<6 & >40
Slope	4–6	6–12	12–16	16–20	<4 & >20
Land use	F	GL	CL	AS	WB&UGS
Aspect	south, Fat	southeast	east, southwest	north, northwest	west, northeast

**Table 5:** Grading of the suitability evaluation indicators for grassland.

Indicators	5	4	3	2	1
Distance to river	<1000	1000–2000	2000–4000	4000–6000	>6000
DEM	>40	30–40	20–30	15–20	<15
Slope	>20	16–20	12–16	4–12	<4
Land use	GL	CL	F	AS&WB	UGS
Aspect	south, Fat	southeast	east, southwest	north, northwest	west, northeast

**Table 6:** Weights of land use suitability evaluation indicators.

Suitability Evaluation Indicators	Weights		
	CL	F	GL
Aspect	0.0736	0.0866	0.0799
DEM	0.1083	0.1733	0.1556
Slope	0.0982	0.1375	0.1233
Land use	0.3035	0.2751	0.3396
Distance to river	0.1468	0.1092	0.0774
Distance to road	0.0827		

**Table 7:** Land use classification accuracy for 2010 and 2020.

Land Use Types	User Accuracy		Producer Accuracy		Overall Accuracy		Kappa	
	2010	2020	2010	2020	2010	2020	2010	2020
AS	0.97	0.99	0.98	0.91	0.91	0.93	0.88	0.91
WB	0.97	0.97	0.93	0.98				
CL	0.97	0.94	0.89	1				
F	0.72	0.85	0.91	0.88				
GL	0.63	0.78	0.78	1				
UGS	0.97	0.96	0.82	0.91				

**Table 8:** The cell number prediction of land use in 2020 and 2030 (Unit: pcs).

Year	AS	WB	CL	F	GL	UGS
2020	19,567,675	10,880,233	1,197,636	5,800,611	1,377,770	8,083,606
2030	20,906,629	8,863,987	423,662	5,957,461	1,320,197	9,435,596

**Table 9:** Model accuracy under different parameter combinations.

Random Variable (a)	Threshold (T)	Simulation Accuracy
0	0.8	86.23%
1	0.8	85.64%
1.5	0.8	84.57%
2	0.8	90.97%
1	0.85	87.80%
1	0.9	88.69%

**Table 10:** Simulated and actual cell number of different land use types in 2020.

Land Use	Actual Number in 2020	Simulated Number in 2020	Difference (%)
AS	19,545,153	20,318,216	3.96
WB	11,159,345	11,081,752	−0.7
CL	1,208,242	1,192,445	−1.31
F	5,724,454	5,686,972	−0.65
GL	1,345,040	1,288,209	−4.23
UGS	7,925,298	7,339,938	−7.39
Kappa	0.87		
Accuracy	90.97%		

**Table 11:** Numbers of the pixels of different land use types in 2010, 2020, and 2030.

Land Use Type	Actual Number in 2010	Actual Number in 2020	Simulated Number in 2030
AS	17,585,184	19,545,153	21,043,750
WB	13,397,157	11,159,345	9,752,536
CL	6,721,090	1,208,242	786,746
F	2,764,562	5,724,454	5,648,884
GL	1,037,747	1,345,040	1,161,655
UGS	5,401,792	7,925,298	8,513,961

## Data Availability

Not applicable.
